# Twenty-Five-Year Trends in Dietary Patterns among Chinese Adults from 1991 to 2015

**DOI:** 10.3390/nu13041327

**Published:** 2021-04-16

**Authors:** Jiguo Zhang, Zhihong Wang, Wenwen Du, Feifei Huang, Hongru Jiang, Jing Bai, Xiaofan Zhang, Bing Zhang, Huijun Wang

**Affiliations:** Department of Public Nutrition, National Institute for Nutrition and Health, Chinese Center for Disease Control and Prevention, Beijing 100050, China; zhangjg@ninh.chinacdc.cn (J.Z.); wangzh@ninh.chinacdc.cn (Z.W.); duww@ninh.chinacdc.cn (W.D.); huangff@ninh.chinacdc.cn (F.H.); jianghr@ninh.chinacdc.cn (H.J.); baijing@ninh.chinacdc.cn (J.B.); zhangxf@ninh.chinacdc.cn (X.Z.); zhangbing@chinacdc.cn (B.Z.)

**Keywords:** dietary patterns, factor analysis, trends, adults

## Abstract

Poor dietary habits have been shown to be associated with a range of chronic diseases and can potentially be a major contributor to non-communicable diseases (NCDs) mortality. We therefore aimed to identify the prevailing dietary patterns among Chinese adults and to evaluate trends in dietary patterns from 1991 to 2015. We used data collected in the China Health and Nutrition Survey (CHNS). Dietary patterns were identified using factor analysis of data from three consecutive 24 h dietary recalls. We studied 29,238 adults aged 18 and above with complete demo-graphic and dietary data. Three distinct dietary patterns were identified: southern (high intakes of rice, vegetables, and pork), modern (high intakes of fruits, dairy products, cakes, cookies, and pastries), and meat (high intakes of organ meats, poultry, and other livestock meat). The southern pattern score decreased (mean ± SD scores in 1991: 0.11 ± 1.13; scores in 2015: −0.22 ± 0.93). The modern pattern score (mean ± SD scores in 1991: −0.44 ± 0.59; scores in 2015: 0.21 ± 1.01) and meat pattern score (mean ± SD scores in 1991: −0.18 ± 0.98; scores in 2015: 0.27 ± 0.91) increased. We observed that China has experienced a shift from traditional dietary patterns to western dietary patterns.

## 1. Introduction

In the past few decades, the national burden of diseases in China has shifted considerably from communicable, maternal, and neonatal diseases to non-communicable diseases (NCDs) [1]. Poor dietary habits have been shown to be associated with a range of chronic diseases and can potentially be a major contributor to NCDs mortality [2,3]. Worldwide, dietary risks account for 12.2% of total disability-adjusted life-years (DALYs) for men and 9.0% of total DALYs for women [4]. Dietary factors are also the leading risk factor for morbidity and mortality among Chinese adults [1,5].

Because dietary intake is so complex, examining and assessing the total diet requires distinct approaches. Traditional dietary analyses have been limited by a focus on the relationship between individual nutrients or foods and diseases [6]. Therefore, dietary pattern analysis has emerged as an alternative, holistic approach that summarizes complex dietary data to render more practical and meaningful information than data on individual foods or nutrients for investigating diet–disease relationships [7,8,9].

China has experienced rapid social and economic development, which have had significant impacts on the traditional Chinese diet [10]. Ongoing monitoring of dietary habits can provide data for nutrition policy development and offer evidence of the impact of the nutrition transition occurring in China. Dietary patterns have been frequently assessed in Chinese studies [11,12,13,14,15,16,17,18]. However, studies that assessed how the patterns change over time are considerably less frequent [19]. Therefore, the objectives of this study were to identify the prevailing dietary patterns among Chinese adults and to evaluate trends in dietary patterns from 1991 to 2015.

## 2. Materials and Methods

### 2.1. Study Design and Subjects

We used data collected in the China Health and Nutrition Survey (CHNS), an ongoing large-scale, longitudinal, household-based survey initiated in 1989. The CHNS used a multistage random-cluster process to draw the sample in nine provinces from northeast to southwest: Heilongjiang, Liaoning, Jiangsu, Shandong, Henan, Hubei, Hunan, Guizhou, and Guangxi. We added three megacities (Beijing, Chongqing and Shanghai) in 2011 and three new provinces (Shaanxi, Yunnan and Zhejiang) in 2015. In total, the CHNS covered 15 provinces that varied in demography, geography, economic development, and public resources. The detailed design and sampling have been reported elsewhere [20,21].

For this study, we used data from nine waves of the survey conducted in 1991, 1993, 1997, 2000, 2004, 2006, 2009, 2011, and 2015. We excluded pregnant or lactating women, those having implausible energy intakes (<800 kilocalories (kcal) per day or >6000 kcal for men and <600 kcal or >4000 kcal for women), and those having unrealistic demographic, socioeconomic, anthropometric, and dietary data. Finally, our analysis included 29,238 adults aged 18 and above with 82,162 complete observations. The study was approved by the Institutional Review committees of the University of North Carolina at Chapel Hill and National Institute for Nutrition and Health, Chinese Center for Disease Control and Prevention. Participants provided their written, informed consent.

### 2.2. Dietary Measurements

Dietary intake was assessed with three consecutive 24 h dietary recalls (2 weekdays and 1 weekend day) in each wave of the CHNS. The participants were asked to report the kinds and amount of the food and beverage items that they consume both at home and away from home during the previous 24 h. Trained interviewers used standard forms to administer the name of food, amount, type of meal and place of consumption of all food items during the 24 h of the previous day in a household interview. The average intake of the three recalls was used for each individual.

### 2.3. Other Relevant Variables

A general questionnaire collected participants’ age, education, living areas, and cigarette smoking habits. Anthropometrical measurements were collected at community or village by well-trained health workers who followed a reference protocol recommended by the World Health Organization. Body mass index (BMI) was calculated using height and weight measurements with one decimal place. In the present study, overweight/obesity was defined as BMI ≥ 24 kg/m^2^ [22].

### 2.4. Statistical Analysis

Factor analysis was conducted to derive food patterns on the basis of 18 foods or food groups (Table S1) of the Chinese Food Composition Table [23]. Mean intake (g/d) was used as the input value in the analysis. The factors were rotated by an orthogonal transformation (Varimax rotation function in SAS) to achieve a more simplistic structure with greater interpretability. In considering the number of factors to retain, we evaluated eigenvalues (>1), the scree plots, and the interpretability of the factors to determine which set of factors can most meaningfully describe distinct food patterns. Items were retained in a factor if they had an absolute correlation ≥ 0.25 with that factor. Factor loadings were calculated for each food group across the factors. A factor score was calculated for each subject for each of the factors, in which intakes of 18 food groups were weighted by their factor loadings and summed.

Trends in dietary pattern scores were assessed by linear regression, using dietary pattern score as the dependent variable and survey year as the independent variable. Per-year change in dietary pattern score (i.e., regression coefficient) was also calculated using linear regression. Adjustment was made for age, gender, educational level, BMI, living areas and current smoking. Trend analysis was also conducted after participants were stratified by these variables. Interactions between these variables and survey year were examined by adding the product term of the time and variable of stratification into the linear regression model. All statistical analyses were performed using the SAS software package (version 9.3; SAS Institute, Inc., Cary, NC, USA). Statistical significance was defined as *p* < 0.05.

## 3. Results

### 3.1. The Characteristics of the Participants

The characteristics of the participants in the analysis by survey year are given in Table 1. Over the study period, the percentages of high school and above, overweight/obesity, and the mean age and BMI increased, while the percentage of current smoking decreased.

### 3.2. Dietary Patterns

Factor analysis revealed three dietary patterns. The factor loadings of each pattern after orthogonal rotation are given in Table 2. These three factors explained 28.9% of the variance in total food intake. Factor 1 was characterized by the food items rice, vegetables, and pork, and was named the southern pattern because it represents a typical traditional diet in South China. Factor 2, characterized by high intakes of fruits, dairy products, cakes, cookies, and pastries, was called the modern pattern. The third factor characterized by high intakes of organ meats, poultry and other livestock meat, was thus called the meat pattern.

### 3.3. Trends in Dietary Patterns

The 25 y trends in the three dietary patterns, overall and according to different sociodemographic characteristics, are given in Table 3, Table 4 and Table 5. Positive scores indicate high adherence to the dietary pattern, whereas negative scores indicate low adherence. After adjustment for gender, age category, BMI, education, living areas, and current smoking, the southern pattern score decreased during the study period while the modern and meat pattern scores increased.

For the southern pattern (Table 3), we observed decreasing trends in all subgroups. The trends in pattern score were similar between gender and living areas. Well-educated participants showed a stronger decrease than participants with lower educational level. Additionally, young participants showed a stronger decrease than older groups.

For the modern pattern (Table 4), we observed increasing trends in all subgroups. The trends in pattern score were similar between age groups and gender. Well-educated participants increased their scores more quickly than participants with lower educational levels. Additionally, urban participants showed a stronger increase than rural participants.

For the meat pattern (Table 5), we observed increasing trends in all subgroups. The trends in pattern score were similar between education and gender. Rural participants increased their scores more quickly than urban participants.

## 4. Discussion

The present study identified three distinct dietary patterns among Chinese adults: southern, modern, and meat. During the period 1991–2015, the southern pattern score decreased while the modern and meat pattern scores increased, suggesting a westernization of dietary patterns in China.

The traditional Chinese diet includes large amounts of cereals and vegetables and small amounts of animal-source foods [10,24]. Because of the agriculture in the area, people living in southern China are more likely to eat rice as a staple food with dishes. We therefore labelled the pattern with high intakes of rice, vegetables and pork as southern, which represents a traditional dietary pattern. Other studies also have reported the similar dietary pattern in the Chinese population [19,25,26]. It was found that the southern pattern had higher tracking, which means the structure of pattern remained stable over time [19]. The decrease in the southern pattern score is in agreement with the consumption of cereals and vegetables that have been in decline over the past decades [27,28].

The modern pattern in the present study had high loadings mostly for snacks, including fruits, dairy products, cakes, cookies, and pastries. The modern pattern scores increased in all subgroups, which suggests that participants are consuming more fruits, dairy products, and packaged foods. In China, fruit has become one of the most popular snack items [10]. However, the intakes of fruits and dairy products were still at the low level, far below recommendations of the dietary guidelines for Chinese residents [28,29]. Because of the fast pace of modern life, people’s demand for convenience foods has become more and more pressing. At the same time, the modern food system has spawned the appearance of small convenience stores or grocery stores in communities throughout China [30]. As a result, people have more access to an array of packaged foods, which we would expect to become a major component of the diet within the next decade.

The consumption of meat among Chinese adults has increased rapidly, and the trend appears to continue [29]. This shift in eating habits could be explained by the decline in the price of meat as well as by an increase in personal income [31]. Rural residents tended to show a stronger increase in the meat pattern score than urban residents, a finding consistent with a previous research [29]. The meat pattern is most common in western countries, which are characterized by a particularly high intake of processed meats and red meats [32]. The most popular meat in China is pork, where fatty fresh pork has already become the main component of total meat intake [29,33]. In addition, consumption of poultry and other livestock meat are increasing [10]. Moderate amounts of meat remain an important source of iron and many other micronutrients, but larger amounts can have adverse health effects [10]. In recent years, Chinese adults, especially urban residents, have consumed meat at much higher levels than recommended, posing potential risks for long-term health.

Our study has several limitations. First, even though the survey captured different demographic and geographic areas, it still did not generate nationally representative results. Second, the 24 h dietary recall method cannot evaluate long-term dietary intake; as a result, the three components explained only 28.9% of the variance in the foods. Third, the study did not analyze factors such as lifestyle habits that may affect the trends. Despite these limitations, to the best of our knowledge, this is the first study to report these dietary trends over a twenty-five years period in such a large sample, when China is in a rapid economic and social development.

## 5. Conclusions

In conclusion, we observed that China has experienced a shift from traditional dietary patterns to western dietary patterns with a decrease in cereals and vegetables consumption and an increase in meat and packaged foods consumption. During the transition of dietary pattern, Chinese people have greater access to varied sources of nutrition. Despite this, China still faces challenges brought by the attraction of an unhealthy diet. Our findings may have important public health implications for the prevention of NCDs like obesity, diabetes, and heart disease. On the other hand, it might be an important issue in nutritional education to draw attention to the unhealthy dietary patterns.

## Figures and Tables

**Table 1:** Characteristics of the participants of the CHNS from 1991 to 2015.

	1991	1993	1997	2000	2004	2006	2009	2011	2015	P for Trend
Sample size	7494	7435	7844	8798	8411	8412	8805	11449	13514	
Female, *n* (%)	3951 (52.7)	3895 (52.4)	4002 (51.0)	4557 (51.8)	4364 (51.9)	4405 (52.4)	4571 (51.9)	5989 (52.3)	7321 (54.2)	0.0078
Age (years)	41.5 ± 15.6	42.6 ± 15.7	43.7 ± 15.6	45.6 ± 15.3	48.5 ± 15.1	49.8 ± 15.0	50.7 ± 15.2	51.2 ± 15.0	52.5 ± 14.7	<0001
High school and above, *n* (%)	1187 (15.8)	1212 (16.3)	1451 (18.5)	1930 (21.9)	2041 (24.3)	2239 (26.6)	2157 (24.5)	3745 (32.7)	4704 (34.8)	<0001
BMI (kg/m^2^)	21.7 ± 2.8	21.8 ± 2.8	22.3 ± 3.0	22.8 ± 3.1	23.0 ± 3.3	23.2 ± 3.2	23.3 ± 3.3	23.7 ± 3.4	24.0 ± 3.4	<0001
Overweight/obesity, *n* (%)	1441 (19.2)	1544 (20.8)	2051 (26.1)	2874 (32.7)	3027 (36.0)	3113 (37.0)	3497 (39.7)	5051 (44.1)	6510 (48.2)	<0001
Rural, *n* (%)	4786 (63.9)	5025 (67.6)	5328 (67.9)	5952 (67.6)	5728 (68.1)	5738 (68.2)	6053 (68.8)	7515 (65.6)	8240 (61.0)	<0001
Current smoking, *n* (%)	2526 (33.7)	2346 (31.5)	2385 (30.4)	2609 (29.6)	2421 (28.8)	2286 (27.2)	2502 (28.4)	3066 (26.8)	3127 (23.1)	<0001

Values are means ± standard deviations unless otherwise indicated. For categorical variables, a Mantel–Haenszel chi-square test was used; for continuous variables, a linear trend test was used with the survey year as a continuous variable in linear regression. Defined based on BMI (kg/m^2^): ≥24 for overweight/obesity.

**Table 2:** Factor-loading matrix for dietary patterns identified by factor analysis among Chinese adults.

	Factor1	Factor2	Factor3
Rice	0.82		
Vegetables	0.52		−0.26
Pork	0.39		0.37
Fish and seafood	0.28	0.26	0.28
Other cereals	−0.44		−0.28
Wheat	−0.68		−0.26
Fruits		0.64	
Dairy products		0.58	
Cakes, cookies and pastries		0.53	
Eggs		0.41	
Nuts and seeds		0.35	
Legumes		0.32	
Fungi and algae		0.31	
Fast foods	−0.27	0.29	
Organ meats			0.43
Poultry			0.41
Other livestock meat			0.39
Starchy roots and tubers			−0.57
Variance explained (%)	11.8	10.7	6.4

Absolute factor loadings ≥0.25 are presented for simplicity.

**Table 3:** Trends for the southern pattern score among Chinese adults from 1991 to 2015.

	1991	1993	1997	2000	2004	2006	2009	2011	2015	P for Trend	Per-Year Change
Sample Size	7494	7435	7844	8798	8411	8412	8805	11449	13514		β ± SE (Standard Errors)
Overall	0.11 ± 1.13	0.13 ± 1.08	0.02 ± 1.06	0.03 ± 0.94	0.07 ± 0.99	0.01 ± 0.96	−0.03 ± 0.95	0.02 ± 1.01	−0.22 ± 0.93	<0001	−0.006 ± 0.000
Gender											
Male	0.20 ± 1.17	0.22 ± 1.11	0.08 ± 1.13	0.10 ± 1.00	0.14 ± 1.04	0.08 ± 1.02	0.04 ± 1.01	0.09 ± 1.01	−0.14 ± 1.01	<0001	−0.004 ± 0.000
Female	0.03 ± 1.09	0.05 ± 1.04	−0.03 ± 0.98	−0.03 ± 0.88	0.01 ± 0.93	−0.05 ± 0.89	−0.09 ± 0.88	−0.04 ± 0.87	−0.28 ± 0.85	<0001	−0.007 ± 0.000
P-interaction										<0001	
Age (years)											
18–44	0.17 ± 1.15	0.18 ± 1.09	0.05 ± 1.06	0.06 ± 0.95	0.08 ± 0.98	0.03 ± 0.97	−0.01 ± 0.96	0.05 ± 0.95	−0.19 ± 0.96	<0001	−0.007 ± 0.000
45–59	0.09 ± 1.11	0.14 ± 1.10	0.06 ± 1.10	0.09 ± 0.97	0.14 ± 1.01	0.07 ± 0.96	0.01 ± 0.96	0.05 ± 0.96	−0.18 ± 0.95	<0001	−0.006 ± 0.000
60+	−0.13 ± 1.02	−0.06 ± 0.96	−0.13 ± 0.95	−0.13 ± 0.85	−0.04 ± 0.96	−0.09 ± 0.92	−0.10 ± 0.91	−0.05 ± 0.91	−0.27 ± 0.87	0.0016	−0.003 ± 0.000
P-interaction										<0001	
Education											
Below high school	0.12 ± 1.16	0.13 ± 1.10	0.01 ± 1.09	0.02 ± 0.97	0.09 ± 1.02	0.02 ± 0.99	−0.02 ± 0.97	0.06 ± 0.96	−0.17 ± 0.94	<0001	−0.003 ± 0.000
High school and above	0.06 ± 0.95	0.16 ± 0.93	0.05 ± 0.90	0.05 ± 0.84	0.03 ± 0.89	−0.02 ± 0.85	−0.06 ± 0.88	−0.06 ± 0.90	−0.31 ± 0.90	<0001	−0.014 ± 0.000
P-interaction										<0001	
Living areas											
Rural	0.17 ± 1.24	0.17 ± 1.16	0.05 ± 1.13	0.07 ± 1.01	0.12 ± 1.05	0.06 ± 1.02	0.02 ± 0.99	0.06 ± 0.96	−0.13 ± 0.96	<0001	−0.004 ± 0.000
Urban	−0.01 ± 0.88	0.05 ± 0.87	−0.04 ± 0.88	−0.05 ± 0.78	−0.03 ± 0.84	−0.09 ± 0.80	−0.12 ± 0.84	−0.06 ± 0.91	−0.35 ± 0.87	<0001	−0.009 ± 0.000
P-interaction										<0001	

Data are presented as mean ± SD. Adjustment was made for age, gender, educational level, BMI, living areas, and current smoking. A linear trend test was used with the survey year as a continuous variable in linear regression.

**Table 4:** Trends for the modern pattern score among Chinese adults from 1991 to 2015.

	1991	1993	1997	2000	2004	2006	2009	2011	2015	P for Trend	Per-Year Change
Sample Size	7494	7435	7844	8798	8411	8412	8805	11449	13514		β ± SE
Overall	−0.44 ± 0.59	−0.44 ± 0.58	−0.31 ± 0.67	−0.25 ± 0.71	−0.12 ± 0.86	0.05 ± 1.04	0.13 ± 0.99	0.67 ± 1.30	0.21 ± 1.01	<0001	0.032 ± 0.000
Gender											
Male	−0.41 ± 0.62	−0.42 ± 0.60	−0.28 ± 0.70	−0.22 ± 0.74	−0.11 ± 0.85	0.05 ± 1.05	0.14 ± 1.00	0.67 ± 1.30	0.20 ± 1.00	<0001	0.030 ± 0.000
Female	−0.46 ± 0.56	−0.46 ± 0.56	−0.34 ± 0.64	−0.27 ± 0.68	−0.13 ± 0.88	0.04 ± 1.04	0.13 ± 0.99	0.68 ± 1.30	0.23 ± 1.02	<0001	0.034 ± 0.000
P-interaction										0.9860	
Age (years)											
18–44	−0.43 ± 0.58	−0.44 ± 0.58	−0.31 ± 0.66	−0.27 ± 0.65	−0.14 ± 0.82	0.04 ± 1.02	0.13 ± 0.96	0.75 ± 1.30	0.19 ± 0.94	<0001	0.034 ± 0.000
45–59	−0.44 ± 0.56	−0.43 ± 0.60	−0.30 ± 0.67	−0.24 ± 0.73	−0.11 ± 0.86	0.06 ± 1.05	0.16 ± 0.99	0.68 ± 1.29	0.22 ± 0.97	<0001	0.032 ± 0.000
60+	−0.42 ± 0.65	−0.48 ± 0.54	−0.32 ± 0.71	−0.20 ± 0.82	−0.08 ± 0.95	0.03 ± 1.07	0.10 ± 1.04	0.59 ± 1.33	0.24 ± 1.11	<0001	0.031 ± 0.000
P-interaction										<0001	
Education											
Below high school	−0.48 ± 0.54	−0.49 ± 0.52	−0.37 ± 0.60	−0.31 ± 0.64	−0.23 ± 0.75	−0.07 ± 0.94	0.02 ± 0.91	0.40 ± 1.14	0.01 ± 0.86	<0001	0.029 ± 0.000
High school and above	−0.19 ± 0.73	−0.20 ± 0.78	−0.04 ± 0.88	−0.01 ± 0.87	0.23 ± 1.07	0.37 ± 1.22	0.50 ± 1.15	1.23 ± 1.43	0.59 ± 1.14	<0001	0.041 ± 0.000
P-interaction										<0001	
Living areas											
Rural	−0.50 ± 0.54	−0.49 ± 0.53	−0.40 ± 0.57	−0.34 ± 0.63	−0.24 ± 0.75	−0.08 ± 0.96	0.02 ± 0.90	0.46 ± 1.20	−0.03 ± 0.80	<0001	0.027 ± 0.000
Urban	−0.33 ± 0.64	−0.35 ± 0.67	−0.13 ± 0.82	−0.06 ± 0.82	0.13 ± 1.01	0.33 ± 1.15	0.38 ± 1.13	1.08 ± 1.40	0.60 ± 1.18	<0001	0.043 ± 0.000
P-interaction										<0001	

Data are presented as mean ± SD. Adjustment was made for age, gender, educational level, BMI, living areas and current smoking. A linear trend test was used with the survey year as a continuous variable in linear regression.

**Table 5:** Trends for the meat pattern score among Chinese adults from 1991 to 2015.

	1991	1993	1997	2000	2004	2006	2009	2011	2015	P for Trend	Per-Year Change
Sample Size	7494	7435	7844	8798	8411	8412	8805	11449	13514		β ± SE
Overall	−0.18 ± 0.98	−0.13 ± 1.01	−0.08 ± 1.04	0.01 ± 0.97	−0.06 ± 1.04	−0.05 ± 1.01	0.03 ± 0.96	−0.00 ± 1.00	0.27 ± 0.91	<0001	0.012 ± 0.000
Gender											
Male	−0.18 ± 1.04	−0.11 ± 1.08	−0.09 ± 1.11	0.01 ± 1.04	−0.06 ± 1.11	−0.05 ± 1.07	0.05 ± 1.03	0.04 ± 1.08	0.31 ± 0.98	<0001	0.014 ± 0.000
Female	−0.18 ± 0.92	−0.15 ± 0.95	−0.08 ± 0.96	0.01 ± 0.91	−0.07 ± 0.97	−0.06 ± 0.95	0.01 ± 0.89	−0.04 ± 0.92	0.24 ± 0.84	<0001	0.011 ± 0.000
P-interaction										0.4804	
Age (years)											
18–44	−0.21 ± 1.01	−0.15 ± 1.05	−0.08 ± 1.08	0.01 ± 1.02	−0.01 ± 1.11	−0.01 ± 1.05	0.10 ± 1.00	0.10 ± 1.06	0.41 ± 0.93	<0001	0.016 ± 0.000
45–59	−0.18 ± 0.99	−0.17 ± 1.00	−0.16 ± 1.03	−0.03 ± 0.94	−0.11 ± 1.03	−0.09 ± 1.05	−0.00 ± 0.98	−0.02 ± 1.01	0.24 ± 0.94	<0001	0.011 ± 0.000
60+	−0.09 ± 0.84	0.00 ± 0.87	0.02 ± 0.88	0.07 ± 0.87	−0.09 ± 0.92	−0.07 ± 0.89	−0.01 ± 0.86	−0.09 ± 0.90	0.17 ± 0.82	<0001	0.006 ± 0.000
P-interaction										0.2543	
Education											
Below high school	−0.24 ± 0.98	−0.20 ± 1.02	−0.19 ± 1.02	−0.09 ± 0.95	−0.18 ± 1.03	−0.17 ± 0.98	−0.06 ± 0.94	−0.13 ± 0.98	0.14 ± 0.88	<0001	0.012 ± 0.000
High school and above	0.13 ± 0.89	0.21 ± 0.92	0.35 ± 0.98	0.37 ± 0.97	0.30 ± 0.99	0.27 ± 1.02	0.32 ± 0.96	0.26 ± 1.00	0.53 ± 0.89	<0001	0.013 ± 0.000
P-interaction										<0001	
Living areas											
Rural	−0.39 ± 0.99	−0.34 ± 1.03	−0.30 ± 1.02	−0.16 ± 0.96	−0.23 ± 1.04	−0.19 ± 1.01	−0.10 ± 0.97	−0.10 ± 1.01	0.12 ± 0.91	<0001	0.016 ± 0.000
Urban	0.18 ± 0.84	0.30 ± 0.83	0.37 ± 0.91	0.37 ± 0.91	0.29 ± 0.95	0.24 ± 0.94	0.32 ± 0.86	0.18 ± 0.97	0.51 ± 0.85	<0001	0.006 ± 0.000
P-interaction										<0001	

Data are presented as mean ± SD. Adjustment was made for age, gender, educational level, BMI, living areas and current smoking. A linear trend test was used with the survey year as a continuous variable in linear regression.

## Data Availability

Data sharing is not applicable to this article.

## Supplementary Materials

The following are available online at https://www.mdpi.com/article/10.3390/nu13041327/s1, Table S1: Food groups in the factor analysis.
