# Supplementary material for: Twenty-Five-Year Trends in Dietary Patterns among Chinese Adults from 1991 to 2015
Source: Nutrients. 2021 Apr 16;13(4):1327. doi: 10.3390/nu13041327 (PMC8072541; doi:10.3390/nu13041327)
Supplement: Supplementary file 1 [file nutrients-13-01327-s001.zip › nutrients-1143387-supplementary.pdf]

Table S1 Food groups in the factor analysis

Food or food groups	Foods included in the group
Rice	Round grained rice, long grained rice, and products
Wheat	Wheat flour and products
Other cereals	Corn, barley, millet, and products
Starchy roots and tubers	Potato, sweet potato, starch, and products
Legumes	Soybean, and products
Fungi and algae	Mushroom, kelp, laver
Vegetables	Cabbage, eggplant, carrot, pepper, lettuce, other vegetables
Fruits	Apple, pear, peach, date, grape, watermelon, orange, other fruit
Pork	Pork and pork products
Other livestock meat	Beef, game, lamb, and meat products
Poultry	Chicken, duck, goose
Organ meats	Organ meats
Fish and seafood	Fish, shrimp, crab, shellfish
Dairy products	Milk, yogurt and products
Eggs	Eggs and products
Nuts and seeds	Walnut, almond kernel, peanut, and others
Cakes, cookies and pastries	Cakes, cookies and pastries, bread, biscuit
Fast foods	Convenience food, hamburger, pizza, sandwich, french fries
